# Factors associated with negative conversion of viral RNA in hospitalized children infected with SARS-CoV-2 Omicron variant in Shanghai, China: a retrospective analysis

**DOI:** 10.1186/s12879-023-08223-x

**Published:** 2023-04-26

**Authors:** Yan Yang, Yannan You, Yazun Liu, Lina Geng, Lirong Huang, Huan Zhou, Xiang Piao, Xiao Liu, Mingyun Wu, Yajuan Wang, Lili Zhou, Peng Wang, Shiping Shen, Mingge Hu, Zhaopeng Han, Zheng Xue

**Affiliations:** Department of Pediatrics, Shanghai Municipal Hospital of Traditional Chinese Medicine, Shanghai University of Traditional Chinese Medicine, 274 Zhijiang Middle Road, Shanghai, 200040 China

**Keywords:** Children, Vaccination, Omicron SARS-CoV-2 variant, Negative conversion

## Abstract

**Objectives:**

This study aimed to identify the related risk factors and potential predictors of SARS-CoV-2 RNA negative conversion by describing the dynamics of viral shedding in infected children admitted to two hospitals from Shanghai during the Omicron variant outbreak.

**Methods:**

This retrospective cohort included laboratory-confirmed cases of SARS-CoV-2 infection from Shanghai between March 28 and May 31, 2022. Clinical characteristics, personal vaccination, and household vaccination rates were collected through electronic health records and telephone interviews.

**Results:**

A total of 603 paediatric patients confirmed to have COVID-19 were included in this study. Both univariate and multivariate analyses were performed to filter independent factors for the duration to viral RNA negative conversion. Data on the redetection of SARS-CoV-2 in the patients after they showed negative results on the RT‒PCR test (intermittent negative status) were also analysed. The median duration of virus shedding was 12 (interquartile range, IQR: 10–14) days. The severity of clinical outcome, personal vaccination-2doses, household vaccination rates, and abnormal defecation were factors indecently affecting negative conversion of SARS-CoV-2 RNA, suggesting that patients who had abnormal defecation or with more severe conditions would have delayed virological clearance, while patients who previously had 2 doses of vaccination or had higher household vaccination rates would have accelerated virological clearance. Loss of appetite (odds ratio (OR): 5.343; 95% CI: 3.307–8.632) and abnormal defecation (OR: 2.840; 95% CI: 1.736–4.645) were significantly associated with intermittent negative status.

**Conclusion:**

These findings could provide clues for the early identification of paediatric patients with prolonged viral shedding and could enrich the evidence for the development of prevention and control strategies, especially vaccination policies for children and adolescents.

## Introduction

Severe acute respiratory syndrome coronavirus 2 (SARS-CoV-2) has been evolving and forming new variants since its emergence in 2019. In the past two years, COVID-19 triggered by SARS-CoV-2 has become a global pandemic, causing severe morbidity and mortality. As of October 14, 2022, the number of COVID-19 cases globally exceeded 620 million, and the number of deaths exceeded 6.54 million [1].

Based on the different virulence and risk factors for SARS-CoV-2 variants, the World Health Organization (WHO) has divided COVID-19 variants into four categories: variants of concern (VOC), variants of interest, and variants under monitoring. The four VOCs – Alpha (B.1.1.7), Beta (B.1.351), Gamma (p.1) and Delta (B.1.617.2) – spread wildly around the world, causing the outbreak to rebound once again [2]. In November 2021, a new variant, the Omicron (B.1.1.529) variant, was detected by genomic surveillance teams in South Africa and Botswana in Gauteng Province, South Africa, becoming the fifth VOC and the current global dominant strain [3].

The Omicron variant was first imported into Hong Kong on November 27, 2021, and was first detected in mainland China on December 9 [4]. In late February 2022, the Omicron variant, the BA.2.2 subvariant, caused a wave of infections in Shanghai. Although 90% of the population in Shanghai has received at least one dose of COVID vaccination [5], a total of 627,087 cases and 589 deaths were recorded by the Shanghai Municipal Health Commission as of the end of June [6]. Dr. Jeane Cloete and colleagues in South Africa conducted a multicentre observational study [7] and revealed that there was a dramatic increase in the paediatric prevalence rate and the number of hospitalizations, especially children and adolescents 1 year and younger, compared with any other time in the course of the pandemic [8]. Although some positive and stringent measures were taken, such as the vaccination programme for children over the age of three, school closures, quarantine at home and even lockdown in the whole city, the prevalence of COVD-19 in children remained grim.

Since the outbreak of the Omicron variant, many studies focused on the epidemiology, clinical features, imaging findings and treatment of infected patients, especially adult patients, have been rapidly launched. However, few studies have investigated the factors and potential predictors of negative conversion in children infected with Omicron variants. In the current study, we described the dynamics of viral shedding in infected children admitted to hospitals from Shanghai during Omicron variant infection and aimed to identify the related risk factors and potential predictors of SARS-CoV-2 RNA negative conversion by a retrospective analysis.

## Methods

The Ethics Committee of the Shanghai Traditional Chinese Medicine Hospital approved this study, and the requirement for informed consent from the parents was waived, as all data used in the current study were deidentified with no private information (No. 2022SHL-KY-19–02). This study followed the Strengthening the Reporting of Observational Studies in Epidemiology (STROBE) reporting guidelines [9].

### Study population and data collection

This retrospective study included 603 infected children under the age of 18 who were hospitalized in designated hospitals and Makeshift Hospital between March 28 and May 31, 2022.

Members of the Shanghai Center for Disease Control and Prevention (CDC) conducted detailed field investigations on suspected COVID-19 paediatric patients identified by SARS-CoV-2 RNA and antigen screening, and respiratory specimens were collected for retest at the Shanghai CDC. Once diagnosed, the children who were under 7 years old, unattended, with obvious symptoms and serious illness, or with severe underlying diseases were sent to the Designated Hospital for treatment, while the children who were over 7 years old without obvious symptoms were arranged to be sent to the Makeshift Hospital for medical observation or symptomatic treatment.

We have drawn up a standardized form for recording the epidemiological, demographic, clinical, laboratory, treatment and outcome data, which were collected mainly through electronic health records and telephone interviews.

### Laboratory procedures

The Shanghai CDC and other nucleic acid testing laboratories used RT‒PCR targeting the open reading frame 1ab (ORF1ab) and nucleocapsid protein (N) genes to detect SARS-CoV-2 in oropharyngeal, nasal, and nasopharyngeal swabs. A cycle threshold (Ct) value was used to approximately reflect the viral loads in the respiratory tract [10]. In our cohort, all patients underwent nasopharyngeal and oropharyngeal swabs for RT‒PCR testing. For the children with symptoms of pneumonia, progression of disease, or recurrence of underlying disease, chest computed tomography (CT) or X-ray examination, routine blood examination, serum electrolytes and other laboratory tests were performed to assess the condition and assist in diagnosis and treatment.

### Definitions

The date of confirmed diagnosis was the time that a positive specimen was collected from oropharyngeal, nasal, nasopharyngeal swabs [11]. A Ct value < 35 was considered positive, and conversely, a Ct value ≥ 35 was considered negative. It was not until two consecutive negative RT‒PCR results obtained at least 24 h apart, which meant the Ct values of the ORF1ab gene and N gene were higher than 35 and clinical symptoms disappeared, that the infected children could be discharged [12]. The time of virus shedding referred to the period from confirmed positivity to the first two consecutive nucleic acid negative results [13]. Intermittent negative status was defined as patients with redetectable viral RNA after showing a negative RT‒PCR test, with a duration from the first day of showing a negative nucleic acid test until the beginning of consecutive negative tests [14].

### Case classifications

According the Novel Coronavirus Pneumonia (Trial Version 9) released by the National Health Commission & State Administration of Traditional Chinese Medicine on March 15, 2022 [12], infected patients are classified into four levels:① asymptomatic-patients with positive RT‒PCR assay result, no relevant clinical manifestations and positive features on imaging manifestations;② mild-patients with mild clinical symptoms, such as fever, cough and phlegm, imaging manifestations showing no signs of pneumonia;③moderate-patients with clinical symptoms, and imaging manifestations showing pneumonia; and ④ severe-patients with increased respiratory rate, poor mental response, abnormal laboratory tests, serious Imaging manifestations or rapid progression in a short period of time.

In our study, negative conversion of viral SARS-CoV-2, as time-to-event data, was the outcome measure. To detect the independent predictors of RNA negative conversion, a survival analysis was performed. The Kaplan‒Meier univariate analysis was first examined with a total of 18 factors. A multivariate Cox regression model was then performed with the significant factors selected by the Kaplan‒Meier analysis to tease out the independent predictors for RNA negative conversion. The association between independent predictors and negative conversion was quantified by the hazard ratio (HR). As negative conversion of viral RNA is a beneficial event, an HR > 1 would promote virological clearance, whereas an HR value < 1 means that the independent predictor would delay negative conversion. A two-sided *P* value < 0.05 was regarded as statistically significant.

Additionally, we investigated factors associated with intermittent negative status. The Mann‒Whitney U test was first conducted to compare differences between the groups with and without intermittent negative status. Next, the factors with statistical significance (*P* < 0.05) were further analysed using the logistic regression model, and ORs were calculated. The Mann‒Whitney U test was also used to compare the time of negative conversion and the viral load between the patients with and without GI symptoms. Analyses were performed using SPSS software (IBM 26.0).

## Results

### Demographics of study participants

Among the 603 paediatric patients, males accounted for 55.7% (336 patients), and females accounted for 44.3% (267 patients). The average age of the patients was 41.1 months, ranging from 30 days to 16.5 years old. More than a quarter of the infected children were under 12 months of age, and 61.9% were under 3 years of age. Among these, 24.7% (149 cases) of the whole cohort were reported to have underlying diseases, including asthma, allergic rhinitis, eczema, recurrent respiratory tract infections and other serious diseases, such as congenital heart disease, tumours, and surgery. A total of 17.9% (108 patients) of the population was allergic to food, drugs or other contacts. For vaccination, only 12 patients received one dose, and 69 patients received two doses due to their younger age. The average vaccination rate of the families with one dose or more was 55.8%, which was lower than the average level in Shanghai and even lower than the vaccination rate of the elderly over 60 years old [5]. Detailed demographical information is available in Table 1.

**Table 1:** Negative conversion of viral RNA among 603 cases with Univariate analysis

**Factors**	**Count (%)**	**Negative conversion rate (%)**			$\mathbf X^2$^1^	***P***
		**7 days**	**14 days**	**21 days**		
**Sex**						
Male	336(55.7)	9.5	76.8	97.3	2.335	0.126
Female	267(44.3)	9.0	76.4	98.3		
**Age**						
< 3	373(61.9)	3.5	73.2	98.7	32.054	0.000^*^
-5	107(17.7)	14.0	83.2	97.2		
6–11	96(15.9)	25	86.5	99.0		
12–17	27(4.8)	30.0	85.2	88.9		
**BMI**						
Underweight	91 (15.1)	18.7	73.6	95.6	2.485	0.478
Normalweight	277 (45.9)	9.4	77.6	97.8		
Overweight	121 (20.1)	9.9	78.5	99.2		
Obesity	114 (18.9)	10.7	79.8	99.1		
**Underlying conditions**^2^						
No	454(75.3)	11.5	79.7	98.5	2.530	0.112
Yes	149(24.7)	10.7	71.1	95.6		
**Allergic history**						
No	495(82.1)	11.1	77.4	98.0	0.000	0.990
Yes	108(17.9)	12.0	78.7	97.2		
**The severity of clinical outcome**						
Asymptomatic	24(4.0)	54.2	95.8	100	30.425	0.000^*^
Mild	399(66.2)	9.8	78.4	98.7		
Moderate	179(29.7)	8.9	73.7	96.6		
Severe	1(0.2)	0	0	0		
**Fever**						
No	77(12.8)	37.8	84.4	98.7	14.963	0.000^*^
Yes	526(87.2)	7.4	76.6	97.9		
**Cough**						
No	282(46.8)	13.8	75.9	97.5	1.199	0.549
Non-productive cough	215(35.7)	6.5	81.4	99.1		
Productive cough	106(17.6)	14.2	74.5	97.2		
**Sore throat**						
No	554(91.9)	10.8	76.4	98.0	.215	0.022^*^
Yes	49(8.1)	16.3	91.8	98.0		
**Rhinobyon**						
No	549(91.0)	11.5	78.0	98.0	0.301	0.583
Yes	54(9.0)	9.3	74.1	98.1		
**Running nose**						
No	533(88.4)	11.8	77.9	97.9	0.648	0.421
Yes	70(11.6)	7.1	75.7	98.6		
**Appetite**						
Normal	510 (84.6)	12.9	66.0	98.0	3.306	0.069
Abnormal	93 (15.4)	2.2	75.3	97.8		
**Defecation**						
Normal	440(73.0)	13.0	81.6	98.6	13.271	0.000^*^
Abnormal	163(27.0)	6.7	66.9	96.3		
**Personal vaccination**						
Unvaccinated	522(88.6)	7.7	75.1	97.9	71.862	0.000^*^
1dose	12(1.5)	33.3	75	100		
2doses	69(9.9)	34.8	95.7	98.6		
**Household vaccination rates**						
≤ 0.25	58(9.6)	5.2	56.9	98.3	36.019	0.000*
0.26–0.50	230(38.1)	5.7	75.2	99.1		
0.51–0.75	219(36.3)	13.7	80.8	95.9		
0.76–1	96(15.9)	22.9	88.5	100		
**Therapy method**						
Medical observation	145(24.0)	13.1	76.6	98.0	0.173	0.678
Symptomatic treatment	458(76.0)	10.7	77.9	98.0		
**Ct-values of N**						
< 20	74(18.4)	0	75.7	97.3	4.083	0.130
20–30	305(50.6)	5.9	77.7	98.4		
> 30	224(37.1)	22.3	57.4	97.8		
**Ct-values of ORFab**						
< 20	68(11.3)	0	77.9	97.1	4.609	0.100
20–30	292(48.4)	5.8	76.4	98.3		
> 30	243(40.3)	21.0	79.0	97.9		

^*^the difference was statistically significant

^1^$\mathrm X^2$ value and *P* values were calculated by Log rank test

^2^Underlying conditions included Asthma, Eczema, Rhinitis, conjunctivitis, tic disorders, ADHD, diabetes, lung disease, kidney disease, cancer, and congenital disease

### Clinical characteristics of children with COVID-19

Among the whole cohort, 24 patients remained asymptomatic, while the other 579 patients emerged with a wide range of symptoms, including symptoms of the respiratory system, digestive system, nervous system, as well as abnormal body temperature and sensation and so on. The proportion of the children with fever symptoms was 87.2% (526 cases). Since fever might precede RT‒PCR positivity and the electronic medical records of the patients did not provide a detailed record of the preadmission temperature, there was no specific grading of the body temperature of the febrile children. From another point of view, the occurrence of fever symptoms prompts parents to take their children to medical treatment in time, which is beneficial to facilitate the treatment and reduce the spread of the virus. Children with respiratory symptoms accounted for over 57.4% (346 patients), among which cough, sputum production and running nose were the main symptoms, accounting for 53.3% (321 patients), 17.6% (106 patients) and 11.6% (70 patients), respectively. More than 40.3% (243 patients) of the children had different gastrointestinal symptoms, such as abnormal defecation (163 patients, 27.0%) and loss of appetite (93 patients, 15.4%). Comparing the patients with and without gastrointestinal symptoms, we found significant differences in the duration of negative conversion, ORFab value, and N value between the two groups at admission. The median duration of virus shedding was 12 (interquartile range, IQR: 10–15) days in the patients without GI symptoms was 13 (IQR: 11–16) days in the patients with GI symptoms. The median ORFab value and N value of the patients admitted without gastrointestinal symptoms were 27.85 (IQR: 23.37–33.21) and 27.86 (IQR: 23.31–33.93), respectively, but the median ORFab value and N value of the patients admitted with gastrointestinal symptoms were 26.07 (IQR: 21.47–30.74) and 25.78 (IQR: 21.14–30.35), respectively. For the nervous system, 1 patient cleared his throat frequently without sore throat and sputum production, 1 patient maintained reticence all the time, 2 patients were irritable, and 3 patients experienced somnolence during the course of the disease. Unlike adults, only 4 patients had fatigue, and none had a decrease or loss of smell or taste. According to Trial Version 9 [11], the 603 paediatric patients were divided into four different degrees of disease: 24 patients were asymptomatic, 399 patients had mild disease, 179 patients had moderate disease, and only 1 patient had severe disease. All children received symptomatic and supportive care according to the guidelines. Oral or intravenous antibiotics are prescribed only when laboratory tests suggest a risk of bacterial infection or when the children have symptoms of a bacterial infection. A total of 26.2% of the children (158 patients) received interferon α2b therapy, which had a certain effect on improving symptoms and shortening the time of negative conversion. Only 5 children tried a new oral small-molecule drug called Paxlovid, which was Manufactured by Pfizer and approved by the WHO for adults with mild to moderate COVID-19 and children aged 12 and over. There was no way to assess its efficacy. Detailed demographical information is available in Table 1.

### Related factors and potential predictors of negative conversion of SARS-CoV-2 RNA

**Fig. 1:**
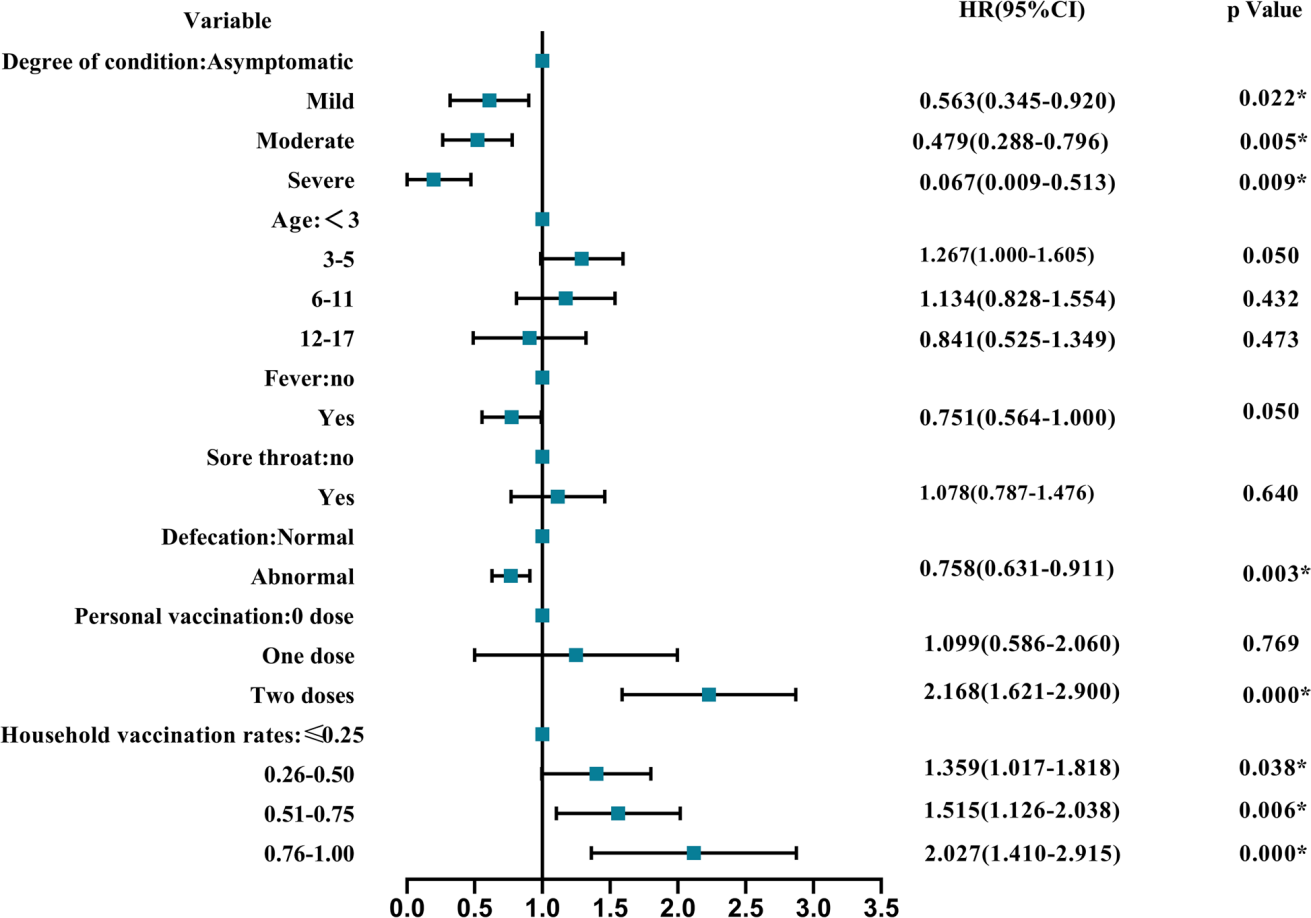
Independent predictors of negative conversion by multivariate Cox regression. Abbreviations: CI, confidence interval

**Fig. 2:**
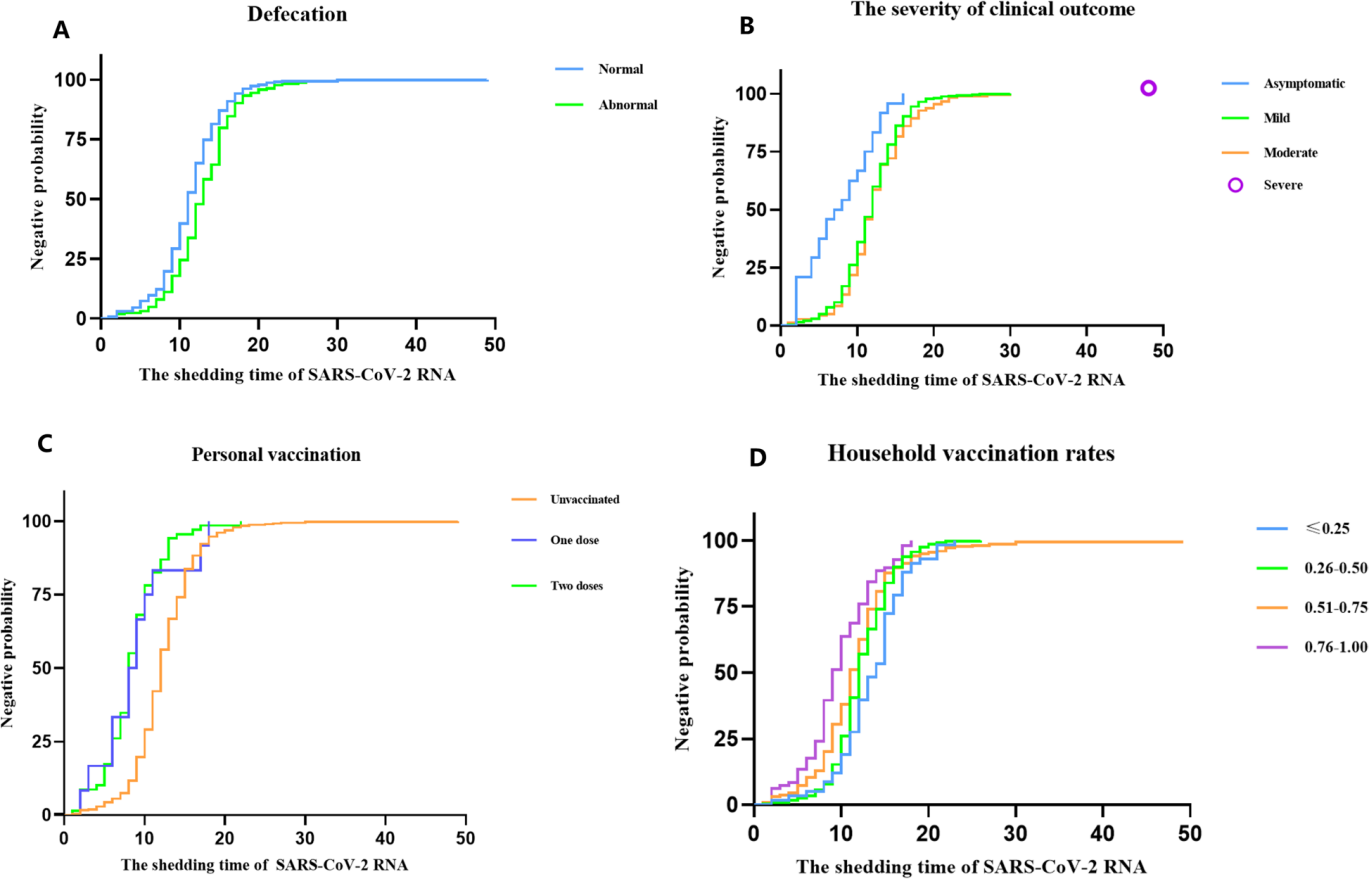
Negative conversion curves according to predictors. **A** Defecation, **B** Personal vaccination, **C** The severity of clinical outcome, **D** Household vaccination rates

According to Trial Version 9 [12], all patients were discharged from the hospital or released from quarantine on the basis of two consecutive negative RT‒PCR assay results at least 24 h apart. At the time of admission, the median CT value for the ORF1ab gene was 27.35 (IQR: 22.94–34.72), and the median Ct value for the N gene was 27.18 (IQR: 22.86–33.12). The median duration of virus shedding in all patients was 12 days (IQR: 9–14), ranging from 2 to 49 days. The rates of RNA-negative conversion within 7 days, 14 days and 21 days among all patients were 11.3% (95% CI: 8.7%-13.8%), 77.6% (95% CI: 74.3%-80.9%), and 98.0% (95% CI: 96.9%-99.1%), respectively. We evaluated the effect of 18 factors on negative conversion of SARS-CoV-2 in 603 cases. The results are summarized in Table 1. As shown above, age, fever, severity of clinical outcome, defecation, sore throat, personal vaccination, and household vaccination rates were significantly related to negative RNA conversion. The significant factors were analysed second by the multivariate Cox regression method (Figs. 1 and 2). The severity of clinical outcome, personal vaccination-2 doses, household vaccination rates, and abnormal defecation were independently associated with negative conversion of viral RNA, suggesting that patients who had abnormal defecation or with more severe conditions would have delayed virological clearance, while patients who had received 2 doses of vaccination or had higher household vaccination rates would have accelerated virological clearance.

### Patients with intermittent negative status

**Fig. 3:**
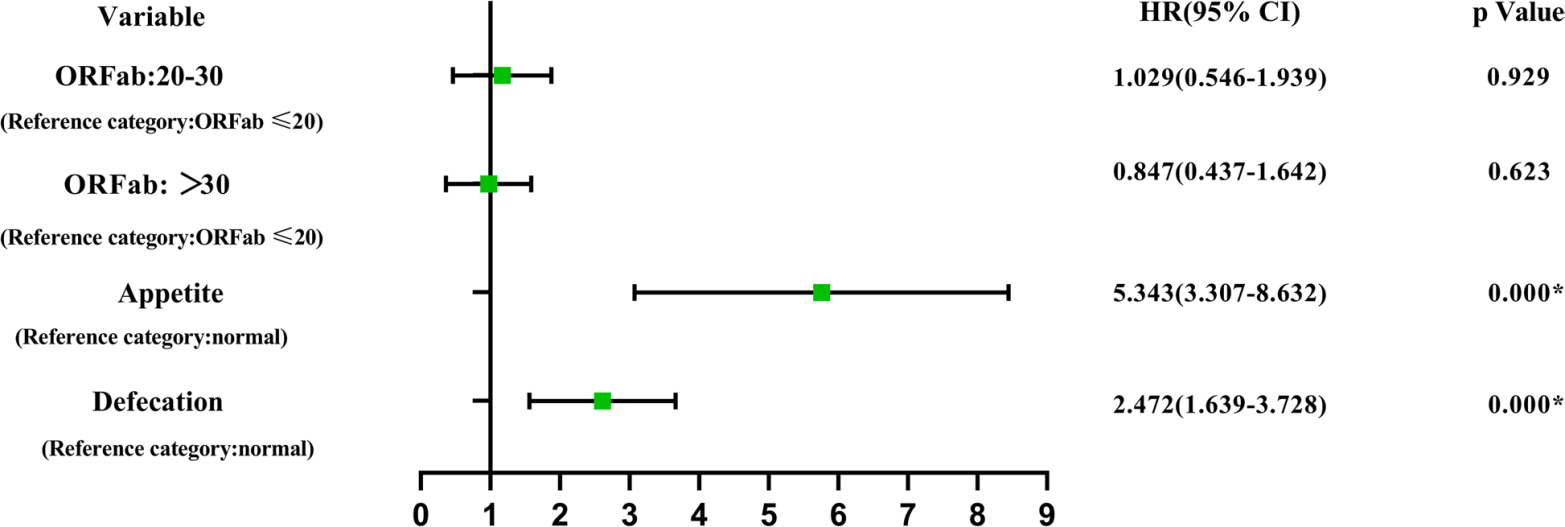
Related factors with intermittent negative status by logistic regression. Abbreviations: CI, confidence interval

When analysing the data for these 603 patients, we found that almost a quarter of the patients fell into a condition in which SARS-CoV-2 was detected again after the first negative nucleic acid. This condition was also observed when SARS-CoV-2 was first discovered in 2019, and researchers defined this state as intermittent negative status. We divided the 603 children into two groups: with intermittent negative status (149 patients) and without intermittent negative status (454 patients) to compare the differences between the two groups. The median time of the children in the intermittent negative status group was 14 days (IQR: 11–15) and that in the nonintermittent group was 11 days (IQR: 9–13). After analysing the variables by the Mann‒Whitney U test, there were three variables, including the Ct value of ORFab, appetite, and defecation, which were significantly different (*P* < 0.05) between the two groups. Further logistic regression with the three variables indicated that loss of appetite (OR: 5.343; 95% CI: 3.307–8.632) and abnormal defecation (OR: 2.840; 95% CI: 1.736–4.645) were positively associated with intermittent negative status (Fig. 3).

## Discussion

In the total study cohort, almost a quarter of the children were infants, and toddlers accounted for 79.6%. The severity of the symptomatic cases was mostly mild (66.2%) to moderate (29.7%), and only one infected child was diagnosed with Niemannpick and underwent liver transplant surgery and required advanced care and treatment. All infected children presented with a wide range of symptoms. The top 5 common symptoms among the symptomatic patients were fever (87.2%, 95% CI: 84.6%-89.9%), cough (52.2%, 95% CI: 49.2%-57.2%), abnormal defecation (27.0%: 95% CI, 23.5%-30.6%), expectoration (17.6%, 95% CI: 14.5%-20.6%), and loss of appetite (15.4%, 95% CI: 12.5%-18.3%). Unlike other respiratory viruses [15], the Omicron variant not only infects the respiratory system but also attacks other systems, such as the gastrointestinal (GI) system, resulting in abdominal pain, nausea, vomiting and abnormal defecation,_,_ and these symptoms are more obvious in patients with Omicron infection, especially in children. Since the children in the designated hospital are too young to accurately describe whether they have subjective symptoms such as nausea and abdominal pain, we have not yet included such indicators.

To the best of our knowledge, this is the first study focused on factors associated with negative conversion of viral RNA of the Omicron variant in paediatric patients. We found that negative conversion of SARS-CoV-2 RNA occurred 11.84 days on average from the first positive RT‒PCR test. It is worth noting that the virus shedding time of child cases in Shanghai is generally longer than that of adult cases infected with SARS-CoV-2 at the same time [16]. We found that abnormal defecation, personal vaccination (2 doses), household vaccination rates, and the severity of clinical outcome were associated with nucleic acid negative conversion by the Kaplan‒Meier method and Cox multivariate regression. The first COVID-19 patient reported in the United States developed gastrointestinal symptoms such as nausea, vomiting, abdominal pain and abnormal defecation during the course of the disease [17], while SARS-CoV-2 was detected in the defecation of this infected person. In subsequent waves of global infections, the highest proportion of people who shed faecal RNA was approximately half of COVID-19 patients, which occurred in the week after infection [18, 19]. In our cohort, we found that patients with gastrointestinal symptoms had longer periods of negative conversion and higher respiratory viral loads on admission. Based on the available evidence, it is very plausible that patients with COVID-19 develop gastrointestinal symptoms attributed to the angiotensin converting enzyme-2 (ACE-2) receptor expressed in epithelial cells of the GI tract, which mediates direct viral entry and infection of the gastrointestinal tissue. COVID-19 patients who shed faecal RNA were more likely to experience gastrointestinal symptoms. It is notable that there are contradictory results in regard to defecation movements. In the Hong Kong cohort, patients with diarrhoea on presentation had higher stool RNA positivity and viral load than those without diarrhoea, while in the USA cohort, patients who shed viral RNA in stool were not more likely to have diarrhoea [18, 19]. In a study by Han et al., patients with gastrointestinal symptoms had a longer duration from symptom onset to virus clearance than patients with respiratory symptoms [20]. This is consistent with our research that children with abnormal defecation had prolonged virological clearance. To the best of our knowledge, our study may be the first to focus on the relationship between GI symptoms and Omicron RNA negative conversion in paediatric patients. Therefore, adequate attention should be given to the occurrence of gastrointestinal symptoms during the epidemic period of the variant.

A global review comparing differences in the incidence and fatality of COVID-19 by SARS-CoV-2 Omicron variant versus Delta variant in relation to vaccination coverage revealed that the decreased incidence and fatality were largely a result of the decreased pathogenicity of Omicron in addition to the increased vaccination coverage during the Omicron epidemic [21]. As pointed out by the WHO in its one-year summary of Omicron on November 25, 2022, the severity of the disease caused by this variant is on average lower than that caused by Delta. The article wrote that many factors may have played a role. For example, the virus mainly causes infection and replication in the upper respiratory tract, due to vaccination and infection, the immunity of people around the world has been steadily improving. The decrease in pathogenicity of the Omicron variant may be related to a structural change in the virus itself that reduces its replication ability, and on the other hand, the protective effect of the vaccination is indispensable. In the group of children, the immune response and physiology were different from adults, such as lower expression of ACE2, elevated baseline IgM targeting coronavirus antigens and stronger early innate antiviral immune responses, which may be the reasons for low prevalence and fewer critical and severe cases in the child population [22–24]. Another large sample retrospective study in Singapore showed that BNT162b2 vaccination reduced the risk of SARS-CoV-2 infection and COVID-19-related hospitalization in children aged 5 to 11 years during a period when the Omicron variant was dominant, which is similar to that of adults who received 3 doses of mRNA vaccination [25]. Thus, vaccination against SARS-CoV-2 still acts as a valuable measure even with a single dose in preventing people from infection and hospitalization. The COVID-19 vaccination program in China was initiated on January 15, 2021, and in June of that year, the age range of COVID-19 vaccination was expanded to include people over 3 years old. In Shanghai [5], a city of 25 million people, children tend to lag behind due to parental concerns, although the overall vaccination coverage is more than 90 percent and the coverage in the 60 s is over 62 percent. The data in our study demonstrated that 2 doses of COVID-19 vaccination can accelerate virus removal. Notably, more than 61.9% of patients under 3 years of age were not authorized to receive vaccination. Although age was not a significant predictor from the multivariate analysis, meaning the factor had no direct effect on time to RT‒PCR conversion, it may indirectly influence viral nucleic acid clearance via the effect of vaccination. We even evaluated the protective effect of household vaccination rates on children on account of the specific importance of households in the context of infectious disease dynamics. It was found that the higher the household vaccination rates, the shorter the time for the children to negative conversion. Similar results were observed during the alpha variant and delta variant pandemics [26]. This should be good news for the formulation of effective and feasible epidemic prevention policies for children and young persons. According to news reports, children aged 6 months to 3 years can be vaccinated with Sinovac starting on August 4 this year in Hong Kong. Further campaigns and actions are needed to promote COVID-19 vaccination for children and adolescents and for their adult family members in mainland China.

Before this epidemic wave, several studies in adult populations reported that a subset of recovered patients who met the criteria for hospital discharge could be retested positive by RT‒PCR [14, 27, 28]. In the current study, 149 patients fell into a condition in which SARS-CoV-2 was detected again after the first negative test result during hospitalization. Comparing the paediatric patients with and without intermittent negative status, we found that GI symptoms, including loss of appetite and abnormal defecation, were correlated with intermittent negative results. Abnormal defecation, as a predictor of negative conversion of SARS-CoV-2 RNA and a sensitive indicator of intermittent negative status, is a crucial element in the whole course of disease and even in the recovery period. Before SARS-CoV-2 mutation, SARS-CoV-2 RNA was continuously detected in faecal samples and anal swabs from the initial batch of confirmed COVID-19 cases [17, 29, 30]. Moreover, clearance of SARS-CoV-2 from the respiratory tract occurred within 2 weeks after the patient’s fever abated, whereas viral RNA remained positive in the faeces of paediatric patients for more than 4 weeks. In addition, viral loads in the faeces might be greater than those in the respiratory specimens [31, 32]. The available data support that the direct role of SARS-CoV-2, cytokine storms, breaks in the intestinal microecological balance and psychological factors may play an important role in infected patients with GI symptoms and positive stool samples during COVID-19 infection [33–36]. Caregivers of children should avoid direct contact with faeces, and precautions need to be taken when aerosol-generating procedures are carried out. It is vital to implement strict hygiene measures after kindergartens and schools reopen to prevent the spread of infections. It might be advisable to expand the testing of SARS-CoV-2 RNA in faecal samples and extend the duration of hospitalization of patients with intermittent negative RT‒PCR results. These findings provide important implications for reducing the retransmission of SARS-CoV-2 and hospital readmissions.

Our study also has several strengths. First, the data included basic information, disease status, clinical characteristics, vaccination status and other information that was as complete as possible. Second, to our knowledge, we demonstrated the importance of gastrointestinal symptoms in children infected with Omicron for the first time. Third, we identified factors associated with negative conversion of SARS-CoV-2 RNA and predictors of intermittent negative status in paediatric patients during the epidemic of the BA2.2 variant. The current study suffers from several limitations. First, data on the patients’ COVID-19 vaccination status, history of allergy, and history of underlying diseases were all based on self-report, and we were unable to verify the information. Second, children in the designated hospital are too young to accurately describe subjective symptoms such as nausea, abdominal pain, and fatigue. As a result, we had to eliminate some of these indices. Third, we did not collect faeces and anal swabs from the paediatric patients for detection, nor did we record the dynamic profiles of viral loads. How the chronological changes in viral loads from faecal samples correlate with disease prognosis merits further investigation.

## Conclusion

In conclusion, this retrospective study identified that the majority of infected children had mild or moderate status, mainly accompanying respiratory and digestive symptoms during the Omicron pandemic in Shanghai. Patients who had abnormal defecation or more severe conditions had delayed virological clearance, while patients who had received 2 doses of vaccination or had higher household vaccination rates had accelerated virological clearance in paediatric patients hospitalized with SARS-CoV-2 infection in Shanghai during the Omicron outbreak. Overall, we hope these findings will provide clues for the early identification of paediatric patients with prolonged viral shedding, enriching the evidence for the development of prevention and control strategies, especially vaccination policies for children and adolescents.

## Data Availability

The used date that support the findings of current study are available from the corresponding author upon reasonable request.
